# Should physical symmetries guide metaphysics? Two reasons why they should maybe not

**DOI:** 10.1007/s13194-023-00525-w

**Published:** 2023-05-15

**Authors:** Cristian López

**Affiliations:** Department of Philosophy, University of Lausanne, CH-1015 Lausanne, Switzerland

**Keywords:** Symmetry, Metaphysics of Science, Fundamental Ontology, Dynamical Laws

## Abstract

Symmetry-based inferences have permeated many discussions in philosophy of physics and metaphysics of science. It is claimed that symmetries in our physical theories would allow us to draw metaphysical conclusions about the world, a view that I call ‘symmetry inferentialism’. This paper is critical to this view. I claim that (a) it assumes a philosophically questionable characterization of the relevant validity domain of physical symmetries, and (b) it overlooks a distinction between two opposing ways through which relevant physical symmetries become established. My conclusion is that symmetry inferentialism loses persuasive force when these two points are taken into consideration.

## Introduction

The connection between symmetries and philosophy has been one of long standing. Ancient Greeks thought of symmetries as closely related to beauty, proportion, and unitary wholes (see, for instance, Plato’s *Timaeus*). Symmetries have also played a crucial role in Christianism as, for instance, in Augustine’s philosophy of divine beauty. The famous Leibniz-Clark correspondence in the eighteenth century is abundant in symmetry-based considerations. It is nonetheless not until the twentieth century that symmetries became a solid scientific concept, shaping the very formulation of the best scientific theories and guiding empirical research. In this trend, philosophers began to draw their attention to symmetries as ways to guide philosophical inquiry on a scientific basis. The connections between physical symmetries, on the one side, and long-standing philosophical issues, on the other, can be traced back to figures such as Albert Einstein, David Hilbert (Ryckman, 2008) and Wigner (1932, 1949) in the twenties and thirties; Weyl (1952), Heisenberg (1975), and Weinberg (1987, 1993) in the second half of the twentieth century; and Sklar (1974), Earman (1989), van Fraassen (1989), and more recently Nozick (2001), in the philosophy of sciences. The list can, of course, be much longer.

In the last decades, philosophers of physics as well as metaphysicians of science have entertained the idea that physical symmetries can also play a more active role in philosophy: they guide philosophers to unveil what’s fundamental in the ontology. Equipped with various inferential mechanisms, physical symmetries were employed as premises to shed light on the nature of space, the nature of time, the natural properties, world’s fundamental structures, and so on. The literature is plenty of examples, in quite varied domains. Whether quantum field theories reveal a “logic of nature”, or whether the world comes equipped with a temporal directionality, or whether the underlying space is homogeneous, are questions to be cast into the mold of physical symmetries. Some have overtly defended the view as a way to do metaphysics of science, for instance, North (2008, 2009, 2013, 2021) and Baker (2010); others have backed their metaphysical views on the basis of symmetry considerations, for instance Ney (2021). This view, which I call ‘symmetry inferentialism’ (SI henceforth), basically consists in understanding symmetries as guides to ontology. The core of the view is an inferential mechanism that takes some physical symmetry as input and delivers a metaphysical conclusion as output. This inferential mechanism, which I call the ‘symmetry-based inference’ (SBI henceforth) is the distinctive mark of SI, although the view and the involved premises can vary from author to author. Be that as it may, the inference has become so a powerful tool for looking into what’s fundamental from scientific and naturalistic means that it would be fair to say that it is a style of reasoning in the field, though not always explicitly.

This paper is overall critical to SI, at least as it stands in the current philosophical literature. In particular, I want to focus on a series of assumptions that are required to put the SBI to work. These assumptions, I submit, are far from trivial and uncontroversial. The first concerns the validity domain of a physical symmetry –*where* and *when* we are entitled to claim that a physical symmetry holds. To determine the validity domain, I argue, we need first to distinguish between the general expression of the laws (or the “covering laws”, in Nancy Cartwright’s vocabulary, Cartwright, 1983) and its instances; and second, to take the former as “metaphysically distinctive”, playing a privileged, more fundamental role. The second assumption involves a tacit tension on which philosophers and physicists have been swinging back and forth in deliberating about the role of physical symmetries –a tension between symmetries as *discovered* and as *stipulated* (Lopez, 2021a, b). Although both approaches are complementary, they pull the role of symmetries in opposite directions. I claim that SBI is hard to justify if the symmetries are stipulated. If I am right on this, then much of the SI’s appealing is lost, diminishing its persuasive force.

The article is organized as follows. In Sect. 2, a characterization of SI as well as the SBI is provided. Section 3 is divided into two parts. SubSect. 3.1 brings to light some of the assumptions required to fence in the validity domain for physical symmetries in the frame of the SBI. SubSect. 3.2 presents two opposing views on symmetries in physics: by stipulation and by discovery. I argue there that the by-stipulation approach is hard to juggle with the SBI. Finally, some concluding remarks.

## From physics to philosophy: Symmetry inferentialism

As I have mentioned above, instances of the SBI have become pervasive in the philosophy of physics and metaphysics of science.^1^ For instance, consider Jill North’s following quote (2008):

In applying any transformation to a theory, we hope to learn about the symmetry of the theory, and *of the world* that theory describes. We do this by comparing the theory with what happens to it after the transformation. If the theory remains the same after the transformation—if it is *invariant* under the transformation—then it is symmetric under that operation. (…) *We conclude* that *a world* described by the theory lacks the structure that would be needed to support an asymmetry under the operation. For example, *from the space-translation invariance* of the laws, *we infer* that *space is homogeneous*, that there is no preferred location in space (North, 2008: 202. Italics mine).

Or, in discussing the origin of the arrow of time, Paul Horwich (1987) distinguishes between three kinds of properties for any series of physical events: particular, general, and nomological properties. The latter are the ones that allow us to infer which is the temporal structure according to physics. He says:

“In addition, the account should help us to understand why the existence of time-symmetric laws is generally taken to guarantee time’s anisotropy” (1987: 39)

Local gauge symmetries are also good examples. Even though local gauge invariances have generally been regarded as simple mathematical redundancy, their astonishing successes have also suggested a more profound interpretation. In this “received view” (Lyre, 2004), the assumption of local gauge invariance “dictates” the kind of interactions and fields we should obtain at some energy regime. For instance, from the demand of local gauge invariance for a free field of electrically charged matter, we obtain the coupling of the Dirac field to an interaction field –in the case of the local $U(1),$ the electromagnetic field. In this way, local gauge invariance serves as guide to discover deep features about the electromagnetic field.

These are cases of SI as a philosophical attitude towards physical symmetries. It takes them as indispensable theoretical posits in our physical theories that entitle us to extract metaphysical claims about the natural world. They thus play a guiding role to discover the fundamental ontology, without being themselves elements in the fundamental ontology. This feature distinguishes SI from other philosophical positions to symmetries in which physical symmetries are placed as properties or even entities of the fundamental ontology (e.g., French, 2014; Schroeren, 2020). In North’s latest book (2021), she says:

“There is a reason for formulating things in terms of structure rather than symmetries, though. Structure is what we are ultimately after (both mathematical structure in the formalism and physical structure in the world), and symmetries are simply an (important) guide to that structure. As mentioned in Chapter 2, symmetries are an indicator of structure, not the structure itself. More importantly, there can be more to the requisite structure than what seems to be indicated by dynamical symmetries” (2021: 73).

SI can therefore be defined as follows:

SI: Symmetries are indispensable as guides *to infer* aspects of the fundamental reality, but they are not themselves aspects of reality.

The distinctive mark, then, is the methodological value of symmetries to infer aspects of natural world at its fundamental level.^2^ This is done by the SBI, in various ways. From an informal and general perspective, the recipe is straightforward. Take a (good enough) physical theory and look at their physical symmetries (e.g., space-translation invariance above). Its symmetries will reveal the structures (or quantities) of the theory that remain unaltered (invariant) under symmetry transformations. Those elements, the argument goes, are to be regarded as fundamental in one’s physics-informed ontology. Thus, philosophical problems gravitating around natural kinds, the nature of space–time, fundamental entities and their interactions may find a suitable solution through instances of the SBI.

From a closer view, we may analyze the different parts involved in the SBI. First, there are premises stating that a physical symmetry (or a set of symmetries) is the case. Although symmetries come in many flavors and shapes (internal vs. external, local vs. global, theoretical vs. observational, geometrical vs. dynamical, and so on), all of them are for the most part *formal* notions that apply to mathematical structures. From a general perspective, physical symmetries are transformations that keep some relevant structure unaltered. In physics, most mathematical structures of interest are sets of differential equations that relate to other mathematical structures (e.g., topological, and differential spaces). In consequence, physical symmetries are transformations that preserve the space of solutions of such sets of differential equations. In this precise sense, physical symmetries are said to be structure-preserving functions that map solutions to solutions. This is the *formal* definition of a physical symmetry. In a classical setting and in the group-theorical language, it can be defined as follows (see Olver, 1993: 92; see also Belot, 2013).

**Formal**_**def**_: Suppose a system $\Delta$ of differential equations involving p independent variables ($x={x}^{1}\dots {x}^{p})$ and q dependent variables ($u={u}^{1}\dots {u}^{q})$. The solutions of $\Delta$ are of the form $u=f(x)$. Let $X={\mathbb{R}}^{p}$, with coordinates $x={x}^{1}\dots {x}^{p}$, be the space representing the independent variables, and let $U={\mathbb{R}}^{q}$, with coordinates $u={u}^{1}\dots {u}^{q}$, represent the dependent variables. A classical symmetry group of the system $\Delta$ will be a local group of transformations, $G$, acting on some open subset $M\subset X\times U$ (kinematically possible fields) in such a way that $G$ transforms solutions of $\Delta$ to other solutions of $\Delta$.

Of course, not any transformation will count as a *physical* symmetry. If this were so, the concept would be trivial and it would always be possible to define a transformation that maps solutions to solutions, augmenting the symmetries of a theory at demand. As Gordon Belot mentions (2013), symmetries are rather hard to come by, so their physical definition should be not too liberal. This in general amounts to imposing further constraints on the formal definition. Some of them can be also purely formal –e.g., for Lie transformations, they must be continuous or smooth; for classical symmetries, the infinitesimal generators must only depend on the independent and dependent variables of the theory, etc. Others can be physical –e.g., Hamiltonian symmetries are required to not only preserve the geometrical structure of the phase space, but also the Hamiltonian. To keep things simple, I assume that a physical symmetry is given by **Formal**_**def**_ plus some physical constraint.

In the light of this, the formal premise(s) in the SBI essentially says a certain formal structure of a physical theory remains unaltered when a symmetry transformation is applied upon it. But, so far, this is just a piece of mathematics plus some minimal physical content. The SBI *somehow* takes a mathematical result as input and gives us a metaphysical conclusion as output, and there is certain consensus that, to achieve that, additional work is needed. Otherwise, the inference would be a “recipe for a disaster” (see Belot, 2013). So, our concept of physical symmetry, in the context of SI and the SBI, not only needs to be narrowed down, but also additional premises are required to mediate between the physical symmetries as formal features of physical theories, and any alleged metaphysical conclusion.

Some authors have pointed out that an epistemic principle needs to be introduced (see  Ismael & van Fraassen, 2003, Dasgupta, 2016): if a physical symmetry holds, there is some structure (or quantity) that becomes superfluous, so it is epistemically advisable going with the most parsimonious structure, “slicing away superfluous structure” (Earman, 1989: 46). This epistemic principle appeals to certain tendency to go with the least structure, serving as a Ochkam’s epistemic razor. Yet, this additional premise is not enough –a more robust, specific concept of symmetry is still in need. There have been various ways to provide such a more robust concept. I do not want to get into details here since it is not the purpose of my argument, but in general these narrowed concepts of symmetries have been related to the concept of objectivity (Nozick, 2001; Weyl, 1952), to undetectable properties (Roberts, 2008, Dasgupta, 2016), to unmeasurable quantities (Ismael & van Fraassen, 2003), or to physical equivalence (Saunders, 2003). Others have been skeptical that the enterprise can be successfully achieved at all (Belot, 2013; Wallace, 2022).

To put all this together, the SBI can be sketched as follows:

**P1.**: Suppose that a set of dynamical equations (*L*) is symmetric under the transformation $\sigma$
**P2.**: If *L* is symmetric under $\sigma$, then it refers to redundant/superfluous structures, to non-objective content, to undetectable properties or unmeasurable quantities.
**P3.**: It is epistemically advisable not to posit those structures, properties or quantities that are superfluous, non-objective, undetectable, or unmeasurable in one’s fundamental ontology.
**CON.**: Therefore, they do not belong to one’s fundamental ontology.

Most common symmetry transformations of interest in philosophy are those that take some spatio-temporal distribution of matter (or structures) and transform it into a new, different distribution. But let us take as an example an internal symmetry, which transforms the value of some “intrinsic” quantity of physical systems. According to the SBI, an internal symmetry implies that the value of some intrinsic quantity can vary freely. Therefore, such a quantity is not part of the fundamental ontology. Baker (2010: 1162–1163) brings up a familiar case in classical electrostatics. The electrostatic potential ($V(x)$) is a quantity which determines the electric field at a point *x*. However, if we transform the gradient of $V(x)$ by adding a constant *c* ($V\left(x\right)\to V\left(x\right)+c$), the gradient remains the same, no difference in the electrostatic force occurs, and therefore no difference to the motion of charged particles. Since the Coulomb’s law is thus left invariant, the change of the value of the electrostatic field is a symmetry. According to the SBI, the electrostatic potential is non-fundamental (however, see Maudlin, 2018 for an alternative view). In Sect. 3.2. I will raise a similar case but focused on local gauge symmetries which can be extended to Baker’s example.

Before proceeding further, five remarks are in order. First, it should be noted that the set of dynamical equations of **P1** usually represents laws of nature which, for the sake of simplicity, are assumed to be the complete laws of the universe. This, of course, does not mean that SBI proponents believe that such a set actually represents the final laws of nature or pertains to the true theory of our world. Nor do they believe that the specific symmetries are essential. Nonetheless, my concern about SBI and SI still stands. The core of my argument is that even if we relativize the claims of SI in this way, the two assumptions to which I want to draw attention are still necessary for SBI to come through. For example, if we assume for simplicity that Newtonian mechanics is the true theory of our world, we still need to assume some ontological fundamentality for the validity domain where Newtonian symmetries hold; and we still need to deal with the tension between by-discovery and by-stipulation approaches. Nothing in my arguments depends on *really* believing that a specific theory is the true theory of the world, nor that a specific symmetry is essential to SBI.

Second, the “quantities” **P2** refers to are those that can vary freely under $\sigma$. Suppose that $\sigma$ stands for Galilean boost (the absolute velocity of all systems increases for a factor *v*). If the (classical) dynamical laws are invariant under Galilean boosts, then we should not posit *absolute* velocities as a feature of our fundamental ontology. Third, the SBI has been also called “the symmetry-to-reality inference” (see Dasgupta, 2016). I believe that such a label is too strong. If, for instance, some property is proved non-objective because it is reference-frame-dependent, this is not enough to declare it unreal *tout court*. It seems to me that the SBI might, at best, help us to identify which structures, properties or quantities are basic (or fundamental) in the ontology of a theory, but it falls short to uphold eliminativism.

Fourth, the SBI aims to reply to *whether-*questions: *whether* space is homogeneous, *whether* time is directed, *whether* a property is natural, fundamental, and so on. But it is worth noticing that physical symmetries may be employed to reply to a different sort of questions in their original theoretical context: *how* space can be homogeneous, *how* this system can be made to evolve forward and backward in time, and so on. These *how-*questions are not, in general, of philosophers’ interest when running a version of the SBI. But they seem to be crucial to understand the role of physical symmetries. I will come back to it in Sect. 3.2.

Fifth, there is a more specific way of reading the SBI. Suppose two symmetry-related experiments yield the same results. This means that there will be some symmetry-variant quantities that can be arbitrarily altered without altering the experimental results. They cannot then be the explanation of the experimental results. Since we should only be committed to those quantities that are necessary to explain our data, we should not believe in a symmetry-variant ontology. Although this is an interesting way to specify the content of SBI, I believe it will ultimately not succeed. Without strong empiricist convictions, it is difficult to justify the premise that we should *only* engage with those quantities that are necessary to explain our data. A first problem is that physical theories as a whole usually account for experimental results; and they are rarely (if ever) formulated in purely empiricist terms. They often involve non-empirical content and non-empirical virtues in order to be formulated and accepted. If such an empiricist reading is adopted, it may end up endorsing some form of skepticism about current physics: we should get rid of all physical content that is strictly unnecessary to explain our data.

More importantly, the reading would be at odds with SI. Most of its proponents want to speak of the structures, entities, or quantities of the fundamental ontology. But it is not clear that we may even need a distinction between a fundamental and a derivative ontology to explain our data –strong empiricism does not need a structured ontology, but (at best) a flat one. We may not even need fundamental entities, quantities, or structures at all. For example, many instances of SI purport to explore which the natural kinds are. But according to the empiricist reading, we can perfectly well explain our data without assuming that there are some unobservable entities, instantiating some intrinsic properties, and that are natural kinds. Indeed, we do not need natural kinds at all to explain experimental results! Note that this specific way to read SBI explicitly demands that we should be *only* committed to those entities, properties or relations that explains our data, which can probably be achieved though some minimal form of phenomenalism and some correlations between observations of experimental results. It is debatable what goes in and what goes out in such a minimal empiricist ontology, but natural kinds are for sure out of the picture. The same goes for space–time and fundamental structures as all these posits may not be considered strictly necessary to explain our data. I do not claim that this interpretation is untenable (it might well be the correct one!). What I claim is that such a reading seems to be at odds with SI. For this reason, I will confine myself to the argument as generally formulated above.

## Where and how: Validity domain and the role of symmetries

Yet, the sketchy representation of SBIs cannot be the whole story. When even a full formulation of the inference is carefully considered, there still seems to be a gap to get bridged –how can it take us from formal and epistemic premises to substantial claims about what’s fundamental? A metaphysical conclusion requires metaphysical premises somewhere. My concern is that there are some metaphysical assumptions that are required by the SBI to work as intended. Even though they go frequently unnoticed, I submit that they do not intervene on the interpretative or epistemic part of the argument, but come in the formal premise(s): What does it mean that a set of laws is symmetric under $\sigma$? Or, better, which assumptions are required for a set of laws to be symmetric under a given transformation when running a version of the SBI? Applications of the SBI have regarded this premise as unproblematic from a conceptual viewpoint, taking it almost for granted. However, it deserves further scrutiny.

The first premise in the SBI (P1) basically establishes that a set of dynamical equations turns out invariant under a symmetry transformation (see **Formal**_**def**_ in Sect. 2), that is, a certain mathematical structure instantiates the property of “being symmetric” under some transformation. This, as was remarked, means that its space of solutions is to be preserved under the transformation. Nonetheless, physical symmetries are hard to come by, since the overwhelming majority of dynamical equations that describe realistic physical situations turn out non-symmetric under many of the symmetry transformations of interest (e.g., geometric, space–time symmetries, or permutation symmetries in quantum theories). Physical symmetries generally hold in special cases, in which a “validity domain”, as it were, has already been fenced in and shielded. From the viewpoint of doing physics, this is harmless –after all, it may well be a theoretical resource for the formulation of physical theories. It may be, for instance, a theoretical resource to make tractable a complex dynamical situation, and then to start building up from it. Yet, in the context of SI and the SBI, the determination of this validity domain acquires a more substantial meaning –it cannot be just a theoretical resource, but it *somehow* needs to be given an ontological meaning. This, I argue in SubSect. 3.1, is far from being conceptually trivial.

There is still another important point. It relates to how a physical symmetry enters P1, that is, how it is introduced in a physical theory to begin with. I have previously mentioned that the SBI offers an answer to *whether*-questions. However, I argue, physical symmetries frequently come in physical theories to reply to *how*-questions. I shed some light on this difference by relying on the distinction between a priori and empirical symmetries (Earman, 1989; Redhead, 1975), or, as it has been more recently put, “by-stipulation” and “by-discovery approaches” to symmetries (Lopez, 2021a, b). The point I make is that SI and the SBI seem to be incompatible with the by-stipulation approach. Since it happens that most physical symmetries of philosophers’ interest have been largely given a by-stipulation construal, the SBI’s appealing may be eroded; and with it, SI’s overall proposal.

### Circumscribing the validity domain: General laws *versus* instances.

For the sake of simplicity, consider a temporal symmetry in the Newtonian formulation of classical mechanics, say, time-reversal symmetry. In this case, the physical symmetry holds (according to Sect. 2) if the Newton Laws’ space of solutions is preserved under time reversal and some relevant physical magnitude remains invariant, for instance, the Hamiltonian. In this context, the symmetry transformation is mathematically implemented by a transformation *T* such that: $T:t\to -t$, $T: x\to x$, $T: v \to -v$. The crucial law here is naturally the Newton Second Law, so I’m going to focus on it. Suppose then a classical point-like particle of mass *m* suspended on a spring. When the spring is released at ${t}_{1}$, the particle experiences an upward movement, so that after a temporal span ${t}_{2}-{t}_{1}=\Delta t$ the whereabout of the particle is some centimeters above its equilibrium position. We assume then that the spring exerts a force of $k.x(t)$ newtons downward (where $k$ is constant). By knowing the initial position of the particle (${x(t}_{0}))$ and its initial velocity (${v(t}_{0}))$, it is straightforward to calculate future positions and velocities through the Newton Second Law

1 $$m.a\left(t\right)+k.x\left(t\right)=0$$

Under certain assumptions about the acting force(s), the equation is proved to be time-reversal symmetric.

Nonetheless, it is worth mentioning that the property of time-reversal symmetry is not a regular feature in Newtonian classical mechanics. Only a few realistic assumptions as air resistance, friction, or imperfect springs are required to obtain a non-symmetric time reversal version of the Newton Second Law. These assumptions can be introduced in the equation through a force that acts in the opposite direction, $-K.v(t)$, where $K$ is a constant. We thus obtain the following equation

2 $$m.a\left(t\right)-K.v\left(t\right)+k.x\left(t\right)=0$$

Which does not remain symmetric under time reversal.

SI recommends us that in order to learn about the properties of time (in a philosophically substantial sense) in a Newtonian world, we should look at the symmetries of the best theory that describes such a world. Both Eq. 2 and Eq. 1 are part of the nomological machinery of Newtonian mechanics, but they clearly pull in opposite directions when introduced in the SBI. The situation is then a bit confusing. It is not clear, prima facie, that a *theoretical* advantage in prioritizing one instead of the other must in consequence be translated into an *ontological* priority. Some (idealized) situations are better described by Eq. 1, whereas others by Eq. 2. Without any ado, it seems we cannot go further than that.

Keith Hutchison (1993, 1995), correctly to my mind, centers on the nature of the forces that intervene, and a fortiori, in the structural relations among the elements of the equation. Whether the Newton Second Law is time-reversal symmetric or not will ultimately depend on the kind force(s) that we are considering in modelling the situation. When forces are too simple (e.g., conservative forces), or when there is no force acting at all, the Newton Second Law comes naturally to be time-reversal invariant; but in the rest of the cases, this cannot be guaranteed. In any case, the problem has simply been moved to a different place: why, if Hutchison is right, should conservative forces be ontologically prioritized over non-conservative ones when plugging the **P1** in the SBI? Once again, the decision is not trivial because the consequences, which depend on it, might greatly diverge.

Let me be more concrete. If we take Eq. 2 for instance and plug it into the SBI scheme, we formulate **P1** in terms of a set of laws that is *not* time-reversal invariant. In adopting the rest of the premises, we may conclude something like the following: Newtonian temporal structure *is* fundamentally anisotropic. However, if we take Eq. 1 and plug it into the SBI, we formulate **P1** in terms of a set of laws that *is* time-reversal invariant. But the conclusion will be the opposite –in assuming that we accept the rest of the premises, Newtonian temporal structure *is* fundamentally isotropic, or directionless. Nevertheless, at this stage, we lack any criterion to select one of the equations instead of the other.

Whoever has gone through the literature already knows that the latter option is the right way to go. But why? Think of the following situation. In the Lagrangian formulation of classical mechanics the dynamics of the physical system is mainly determined by the Lagrangian. So, the symmetries of the Lagrangian are now of our interest. Suppose a single particle in an inhomogeneous potential that depends on positions. The Lagrangian is clearly non-invariant under space displacement. Should we then conclude that space is inhomogeneous and that there are “privileged” positions? This certainly makes little sense. The natural explanation is that the spatial inhomogeneity is merely apparent and has been caused by the sort of interactions that the potential brings about. The asymmetry of time in Newtonian classical mechanics is, mutatis mutandis, apparent in the same way. It follows actually from having focused on the non-fundamental scenario, that is, one on which non-conservative forces intervene.

The take-home message is that these asymmetries are to be related not to properties of the space and time, but to properties of the forces and the potential. If some interactions have the capacity of, as it were, “distorting” spatial relations, or of picking out a temporal direction, then, to explore the genuine properties of space and time, one should abstract away all interactions (or, at least, those that have this distortive capacity). Somehow, those forces and interactions must be ontologically degraded. Hence, the relevant conditions must be phrased differently: “if there were no dissipative forces, if springs were perfect…then the set of laws *L* would be symmetric under $\sigma$”. Therefore, one is led to frame **P1**, and SBI consequently, in a “circumscribed” validity domain, where covering (or general) laws are only considered, where certain kind of forces and interactions (but not others) intervene. That is, SBI requires us to focus on highly idealized domains (e.g., Eq. 1) because they have been previously assumed to be not only theoretically, but also ontologically, more fundamental. This metaphysical decision does not follow from the argument, but it is on the basis of selecting the right content for **P1**.

From a metaphysical point of view, the SBI can thus determine the fundamental ontology through the physical symmetries of that validity domain, while it can also determine the secondary (or supervenient, or emergent) ontology through the symmetry breaks that occur in less idealized domains. In the case of time-reversal symmetry in classical Newtonian mechanics, the metaphysical lesson is that there is no *fundamental* direction of time if the world were Newtonian, but that it is an “emergent” feature that appears when interactions or some forces intervene (e.g., at the level of springs and non-conservative forces). Therefore, the SBI is a guide to the fundamental ontology while also determining what the non-fundamental ontology is.

I do not mean that SI’s rationale is not right or effective. Indeed, it is. But I want to draw the attention towards its assumptions in the frame of SI. *If* SI is to make sense, then it must select the right validity domain to be able to select situations like that of Eq. 1, and to discard cases as those of Eq. 2. In one way or another, certain expressions of the laws must be beforehand chosen as privileged to fill **P1** (e.g., the covering laws, instead of the phenomenological laws). This underlies part of the SBI and cannot be decided on the basis of the physical theory at stake; nor of the SBI itself. There is nothing idiosyncratic in the selected cases either –what goes by a physical theory (e.g., Lagrangian classical mechanics, Newtonian classical mechanics, Quantum Electrodynamics, and so on) involves a massive number of particular laws and models. It is normally required that some general expression of the dynamics must be found to cover all the intended cases of application. That’s why we are given the covering laws as “the” laws of the theory, which are the “schemes” to be filled case by case. And it happens that *these* general or covering laws are those that typically instantiate many of the physical symmetry of interest. But this theoretical machinery, with its internal distinctions, does not necessarily translate into ontological distinctions.

To achieve this, then, an additional assumption is required. It not only requires a sharp distinction between the covering laws and their instances, but that such a distinction be promoted to an ontological distinction as well. If for whichever epistemic or methodological reason covering, or general expressions of the laws are prior, their priority is also an ontological priority –they are ontologically more fundamental. To explain it in other words, in the frame of a SBI, **P1** already bears some non-trivial ontological content –the fact that “a set of laws *L* is symmetric under $\sigma$” entail that (1) a certain validity domain has been circumscribed, (2) that such a validity domain is ontologically prior, (3) that the set of laws *L* describing/governing such a domain are also ontologically prior. Otherwise, the choice between cases like Eq. 1 vs Eq. 2 are unwarranted and the gap between the physical theory and the ontology couldn’t be bridged.

Craig Callender (1995) has made this explicit: when we ask whether some equation is symmetric under some transformation, we ask whether it is *fundamentally* symmetric. We are then directing the question to those laws that are regarded as ontologically prior –the *fundamental* laws. This already draws a sharp line between these and their instances (called ‘phenomenological’). But it also presupposes that the covering laws describe (or govern) matter at some fundamental level, and thereby, their physical symmetries also express a feature of matter at the fundamental level. Of course, this is not an innocuous metaphysical assumption: a plausibly mere theoretical distinction gets translated into the ontology –non-conservative forces, for instance, are metaphysically less fundamental (or even unreal) (see Callender, 1995: 333). Now, it looks more natural what is special about Eq. 1–it is representationally more fundamental because it is metaphysically more fundamental (for discussion, see Hutchison, 1993, 1995). We have already structured the physical ontology in such a way that some forces and interactions were given a fundamental place, whereas others a derivative or emergent one. All these metaphysical assumptions, I submit, are already at play in selecting **P1** in the SBI. But, more importantly, they are needed to connect the formal premise to any metaphysical conclusions. Otherwise, the SBI cannot work as intended.

### Two opposing approaches: By-stipulation and By-discovery

Previous subsection focused on the necessity of circumscribing a validity domain. In so doing, some metaphysical content must enter the SBI through **P1**. In this subsection, I focus on a related, but different aspect: when a validity domain has been already circumscribed, how do we come to know that a physical symmetry holds? And, within a validity domain, which role does the symmetry play? Both questions, as I will show, are interconnected.

Michael Redhead (1975) has distinguished between two views of physical symmetries: the a priori view, “which seeks to derive laws of nature from symmetry principles”, and the empirical view, “which derives symmetry principles from known laws of nature and expresses interesting mathematical properties of such laws” (1975: 80). He adds that a priori symmetries generally tell us something about our knowledge of the world, but not about the world itself. Interestingly, Redhead’s distinction keeps in line with Earman’s:

“The received wisdom about the status of symmetry principles has it that one must confront a choice between the *a posteriori approach* (a.k.a. the bottom up approach) versus the *a priori approach* (a.k.a. the top down approach)”. (Earman, 2004, 1230)

Brading and Castellani (2007) have also pointed that some take symmetries as postulated, guiding theory construction, while others as a consequence of particular laws –like a discovery (2007: 1347). More recently, Lopez (2021a, b) distinguishes between two approaches: by-discovery and by-stipulation. In essence, Lopez’s distinction suggests that while some physical symmetries play a normative role in theory construction (the by-stipulation view), others regard them as a by-product of empirically well-grounded laws (the by-discovery view). The normative status of stipulated symmetries, according to him, makes them a priori and necessary within the context of a physical theory; but, if they are taken as discovered, then their status may be a posteriori and contingent. I will draw on Lopez’s distinction to carry out my argument here.^3^

Why is such a distinction important in the context of SI? My claim is that the persuasive force of the view strongly depends on whether we consider physical symmetries as stipulated or as discovered. I do not mean this to be a knock-down argument; but the point I want to argue for is that SI loses much of its persuasive force if physical symmetries are regarded as normative statements. This normative character derives from their stipulated nature, which pursues some methodological, heuristic, and epistemic goals to construct an acceptable physical theory, and especially, to construct the validity domain of its symmetries. Although a by-discovery view of symmetries would be safe from this criticism, it happens that the majority of physical symmetries that have drawn philosophers’ and metaphysicians’ attention are indeed stipulated symmetries.

My argument then runs as follows:

1. Physical symmetries may be either stipulated or discovered.
2. If physical symmetries are stipulated, then the SBI loses much of its persuasive force.
3. It happens that the majority of the physical symmetries appearing in the SBI are stipulated.
4. Therefore, the SBI loses much of its persuasive force.

Let me argue for each premise one at a time. The first premise is an assumption and, I believe, is not very problematic. I have already shown some textual evidence that such a distinction has been in the air for a while. Note that it is not committed to see both approaches (by-stipulation and by-discovery) as globally exclusive. That is, both can coexist –some physical symmetries may be regarded as stipulated, whereas others as discovered. But, of course, the *same* physical symmetry cannot be construed as stipulated and discovered simultaneously. In individual cases, both approaches exclude each other. The second and third premises are more problematic. The third is in need for concrete cases; the second for an analysis of the cases. Let me defend both with two examples. The first one relates to the stipulation of local gauge invariance and “the gauge argument” (Hetzroni, 2021; Martin, 2002; Teller, 1998), which lies at the core of the construction of interacting field theories^4^; the second one with early general considerations on symmetries in relation to laws of nature.

What do I mean by “the SBI loses much of its persuasive force”? I simply mean that we won’t be equally well disposed to accept the SBI if physical symmetries are viewed as normative, stipulated rules for theory construction. If the SBI loses persuasive force, so does SI. It can be argued that the “persuasive force” of the SBI comes from empirical evidence: physical theories that stipulate some symmetries not only work, but also show us that there are no experimental differences between symmetry-related scenarios (for instance, between Galilean boosted systems). So, one might consider that the normative, stipulated character of symmetries obeys some heuristic or pragmatic goal, but one might also consider that there is actually an empirical basis backing them. This would partially undermine my argument.^5^ I would nonetheless be a bit cautious. Two reasons. First, in general, most physical theories are metaphysically underdetermined, which means that their empirical basis does not settle their ontology. Of course, empirical adequacy is an ingredient in settling an ontology, but it is not the only one –other non-empirical virtues also play a relevant role. Think of the philosophical debate on laws of nature. It is undeniable that the laws of physics of our best physical theories play a central role in the experimental success of physical theories. Yet, the empirical basis does not settle, for instance, whether the laws “govern” behaviors in the fundamental ontology, or “emerge” from dispositions. I believe that physical symmetries (and the SBI) can be assessed similarly. Second, even though empirical adequacy is important, we can have other physical reasons to not take a physical symmetry too metaphysically seriously. A paradigmatic case is the attempts to make Bohmian mechanics compatible with special relativity –Bohmian mechanics can be formulated in a relativistic space–time by introducing a privileged foliation (see Dürr et al., 2014). It is clear that this violates special relativity, but it is not seen as problematic since the goal is to formulate a more general theory that is also empirically adequate.

To be clear, my claim is that if my argument is correct, the SBI loses persuasive force as it stands. This means that it requires some further argumentation to be restored and accepted.

#### The stipulation of local gauge symmetries

Suppose a field $\Psi$ representing electrically charged matter. The action corresponding to the free field (obeying Euler–Lagrange equations) is invariant under a *global* phase transformation, which basically means that the phase transformation,$\Psi \to {e}^{iq\Lambda }\Psi ; \overline{\Psi }\to {e }^{-iq\Lambda }\overline{\Psi }$, does not depend on spatial coordinates but induces a constant phase shift in$\Psi$. This is the so-called global $U(1)$ transformation group.

In the application of the global phase transformation, the $\Lambda$ was constant. But, if the phase transformation is demanded to be *local*, $\Lambda$ becomes now a function of the space coordinates, $\Lambda \left(x\right):\Psi \to {e}^{iq\Lambda (\mathrm{x})}\Psi ; \overline{\Psi }\to {e }^{-iq\Lambda (\mathrm{x})}\overline{\Psi }$. However, the free field Lagrangian is no longer invariant under the local phase transformation. The story of how local phase invariance is restored is well-known –the free field Lagrangian is modified and replaced by an interacting Lagrangian that introduces a field, the gauge potential${A}_{\mu }$, that couples with the matter field in a now interacting Lagrangian. This new interacting Lagrangian becomes invariant under the local phase transformation *if* the gauge potential transforms in a specific way, namely, analogous to how the electromagnetic gauge transformation does, ${A}_{\mu }\to {A}_{\mu }+{\delta }_{\mu }\Lambda$ (which has suggested that the new field ${A}_{\mu }$ represents the electromagnetic potential). To obtain a fully interacting theory, a kinematic term needs to be added to the interacting Lagrangian, which “imbues the vector field [${A}_{\mu }$] with its own existence” (Martin, 2002: S224).

Two points are noteworthy. First, the gauge argument works. It has worked incredibly well to formulate empirically successful interacting field theories and lies at the core of the many gauge theories we adopt in particle physics today (remarkably, quantum electrodynamics) (see Roberts et al., 2021). Second, the gauge argument is very powerful, but it is in companion with other assumptions, as it was well noted. For instance, there is no unique way to modify the Lagrangian to achieve local gauge invariance, but additional constraints must be added as well. It can, for instance, be required that the “minimal modification” must be chosen, which already assumes simplicity. Martin has convincingly argued that, more importantly, re-normalizability plays a crucial role in picking out the minimal modification (Martin, 2003).

Having said that, let us now focus on how local gauge invariance comes in the physics. First, it doesn’t spring fully armed out of nowhere, but it emerges from the *global* $U(1)$ and the consequent failure in localizing it. However, in order to construct local gauge *invariant* theories, it is demanded (or, better, *stipulated*) that the interacting field theory *ought to* be invariant under the local phase transformation. The thus-obtained interacting theory follows, partially but necessarily, from the stipulation of local gauge invariance along with additional assumptions (simplicity, re-normalizability, etc.). These elements are crucial if the gauge argument is meant to work. But are there any grounds for demanding *local* gauge invariance to begin with? The requirement of locality has been widely disputed and argued for differently. Whereas Yang and Mill’s 1954 seminal paper suggests that the demand of locality follows from pursuing a *local* field theory (allegedly incompatible with global phase transformation), others have argued that such a demand is tied to the locality required by special relativity (Ryder, 1996).

Putting aside the reasons, the point is that it is assumed that the theory *must be such that* local gauge invariance holds. That is, the reasons somehow rely on normative criteria to build up an acceptable field theory that largely exceeds mere empirical adequacy (e.g., the requirement of locality to follow the spirit of special relativity depends on assuming inter-theoretical coherence, theoretical correspondence, and systematic unity). But this is not just for the demand of locality, but also for the very demand of invariance (or symmetry) in general. The demand of local gauge *invariance* is not uniquely motivated by empirical adequacy either, but also by general epistemic standards related to what is a good physical theory and which requirements it must meet.^6^ The series of assumptions that intervene on demanding that a physical symmetry be the case is quite complex but involve many methodological assumptions and normative standards that heuristically guide us towards acceptable theories. Of course, empirical adequacy is necessary for any physical theory to succeed, but it is no sufficient –the theory not only has to perform well when it comes to empirical testing, but it must be, previously, an acceptable theory.

The stipulation of physical symmetries, and in this particular case the stipulation of local gauge invariance, plays a crucial role here since it constrains the set of possible physical theories that are to be qualified as “acceptable”. Physical theories that exhibit many physical symmetries are generally simpler, more manageable, and fundamentally, more modally adaptable than their less symmetric competitors. They can cover alternative scenarios with less (potentially arbitrary) structure. For instance, we do not know if reality is temporally directed (fundamentally). But if we can formulate a physical theory in which the direction of time is just a superfluous mathematical parametrization of an evolution, *methodologically* assuming that the form of dynamical law should not change under a change in the mathematical representation, we have formulated a more general, simpler, and explanatory theory. In some cases, physical theories can be formulated in such a general fashion; but sometimes they cannot. What it is crucial here is that generality, simplicity, and explanatory power (or the norms that asses them) should not be mixed with truth (Cartwright, 1983) and further arguments are in order. More importantly, the idea of stipulation is closely in keep with the idea of making explicit the necessary resources we might need to gain explanatory power within certain previously adopted standards of epistemic adequacy. In this way, whether a physical symmetry is the case or not when stipulated has more to do with epistemic standards for evaluating physical theories than to what the world might be like.

#### Symmetries and the possibility of laws of nature

The last paragraph leads to my second example. Eugene Wigner (1949) held that the human capacity of abstracting and idealizing makes science possible. In particular, he referred to the human capacity of devising “artifices” that “permits the complicated nature of the world to be blamed on something which is called accidental, and thus permits him to abstract a domain in which simple laws can be found” (1949: 521). To achieve this, Wigner says, it is essential that equal initial conditions deliver the same results regardless of *when* and *where* the initial conditions are realized, which means that “the absolute position and the absolute time are never essential initial conditions” (1949: 521). Otherwise, laws of nature could not even be formulated.^7^ Wigner is clearly referring to time- and space-displacement invariance, which play a crucial role not only in deriving the laws of nature, but in making them possible by circumscribing an idealized validity domain. Then, the stipulation of time- and space-displacement aligns naturally with a normative construal of symmetries –they not only make what is for a physical explanation be a good and acceptable physical explanation, but also make a law of nature be what it is.

But, if physical symmetries ultimately play a normative role in circumscribing a validity domain in which we can recognize laws of nature, what entitles us to draw a metaphysical lesson from this? Wigner’s comment seems, indeed, to suggest otherwise: physics is not in the business of studying the nature of things, but their regularities; for physics to do so, laws of nature are essential. But the laws of nature (as we conceive and accept them in modern physics) would not be laws of nature *if* they weren’t symmetric under time- and space-displacement. If Wigner is right on this, then some physical symmetries of the laws come in to play a normative-heuristic role, almost definitional of the validity domain. But this can hardly square with SI’s intentions: how may *norms* for theory construction play a paramount role in metaphysical reasoning? To put it differently: *if* they are norms for theory construction, then they do not seem to be the right sort of theoretical resources that may guide us to what is fundamental in the ontology –they have come in the theory as necessary methodological principles for the very theory to be possible as a way to study stable regularities.

To be clear, the problem is not really about epistemic considerations in general (they can of course play a role in metaphysical reasoning), but rather about the methodological, heuristic and epistemic *normative* rules that intervene in theory construction or in guiding the reasoning of a discipline as a whole. They, for instance, impose some standards of acceptability (what is to be a “good” physical theory), which cannot be reduced to mere empirical adequacy and, I believe, should not serve as guide to metaphysics. Wigner’s argument, as I read it, states that symmetries seem to play a role in making laws of nature possible. But the conditions for making something possible are closer to a Neo-Kantian transcendental argument, rather than guides to metaphysics. Of course, Wigner’s view (or any view close to it) can be rejected and arguments should be provided against it. But, *if* it is accepted, then it seems to suggest that symmetries can hardly serve as guides to metaphysics.

Coming back to Wigner’s view, let me address the issue but from a slightly different angle. SI and many instances of the SBI have relied on a hypothetical tight relation between some symmetries of the dynamical laws and the symmetries of space–time. This connection prima facie entitles SI to draw *metaphysical* lessons about the space–time’s structure by looking at the symmetries of the dynamics. As a case, Eq. 1 in my example above bears a “special connection” with the underlying space–time structure because in knowing the symmetries of the former, some properties of the later can be learnt.

This connection is not arbitrary, but it is very well-grounded. It has been explained in many places (see Wigner, 1964, see Hetzroni, 2021 for a more general formulation) but is crystal clear in what John Earman has called “the adequacy criteria” (1989: 46):

**SP1**: Any dynamical symmetry of *T* is a space–time symmetry of *T*
**SP2**: Any space–time symmetry of *T* is a dynamical symmetry of *T*

North (2009) has rephrased Earman’s principles in terms of a unified *methodological* principle: “Physics adheres to the methodological principle that the symmetries in the laws match the symmetries in the structure of the world [i.e., the structure of the space–time]” (2009: 65). I will take for granted that the principle, as it stands, is sound.

Yet, this well-grounded principle is not enough for us to conclude that both symmetries ultimately mirror the real nature of space–time, nor that physical symmetries may guide us to what’s fundamental. First and foremost, the principle is passible of either an epistemic-normative or an ontological reading. In an epistemic-normative reading, the principle just expresses a heuristic guide for theory construction –a good, acceptable physical theory ought to follow this principle as much as it can; or, to put it differently, in the formulation of a physical theory we ought to *heuristically* follow this principle as much as we can. In this light, the principle just serves to assess physical theories, not to bridge the gap between the theory, on the one side, and the ontology, on the other. SI however seems to require an ontological reading for the SBIs to go through; that is, the principle cannot be merely heuristic, but it must be also able to bridge the gap between physical symmetries and the ontology (i.e., between **P1** and **CON**). This is of course possible, but it does not directly follow from the mere existence, or the mere wide acceptance, of the principle. The ontological reading must be explicated and added as an additional assumption –the connection at the level of the theory is also a connection at the level of the ontology.

But is an ontological reading of the principle even plausible? Even though I don’t mean it to be impossible, it is hard to justify. Earman’s formulation of the principles is twofold: it goes from dynamical symmetries to space–time symmetries *and* the other way around. Notwithstanding this, SI frequently employs the principle in only one direction –the inference goes from properties of the laws to properties of the space–time (or, more generally, to the geometrical structure). To say it differently, the SBI hardly ever employs the properties of space–time as a premise to get properties of the laws as outputs, but the opposite: the properties of space–time (or the kind of geometry of the fundamental space) are almost always the conclusion of some versions of the SBI. Not only is this preference for one direction absent in Earman’s version of the principle, but it has also been held the other way around. To come back to Wigner, he says that.

“The postulate of the invariance with respect to displacement in space and time disregards this possibility and its application on the cosmological scale virtually *presupposes* a homogeneous and stationary universe” (1949: 521. Italics mine)

For Wigner, the postulation of a space–time symmetry for the laws follows from having assumed that the space was homogeneous, that is, that there are no absolute positions in the space! If we think this through in the light of the SBI, it seems that **CON** has already sneaked in **P1** –it is a necessary assumption to postulate that the dynamical structure in question instantiates the relevant symmetry. In simpler words, we have just gotten what was already there. But this is not just Wigner’s own outlandish view. In the formulation of Bohmian Mechanics, Dettlef Dürr and Stephan Teufel (2009) hold something similar. They say in passing:

“Let us close with a final remark on time-reversal invariance. One should ask why we are so keen to have this feature in the fundamental laws when we experience the contrary. Indeed, we typically experience thermodynamic changes which are irreversible, i.e., which are not time reversible. The simple answer is that *our platonic idea (or mathematical idea) of time and space is that they are without preferred direction*, and that the “directed” experience we have is to be explained from the underlying time symmetric law” (2009: 47. Italics mine)

To be emphatic, if the properties of the space–time are an assumption in the explanation of why a dynamical structure exhibits the symmetries it does, then the SBI is severely eroded, risking circularity.^8^ Of course, there are plenty instances of adopting the principle in the opposite direction, as SI does. But I’m not saying that there is just one right direction, but that there seems to be none. If this is so, the burden of the proof is on SI: why should the principle be read in one direction, rather than in the other?

None of these problems arises if an epistemic-normative reading of the principle is given. In that case, it is clear that they are necessary assumptions to, on the one hand, formulate general and far-reaching laws of nature, and to, on the other, keep the dynamical and the geometrical structure on a par. That the laws of nature turn out symmetric under, for instance, time- and space-displacement sheds no more light on the structure of the underlying space and time than was already there when it was stipulated that such laws should be symmetric. In this context, an ontological reading seems not only to be problematic because the risk of circularity, but also because is at the edge of a categorical mistake –norms and heuristic (or even regulative) principles are not the sort of things that we want to take metaphysically seriously, at least when it comes to tailor the scientific ontology.

The difference between *whether*- and *how*-questions that I mentioned earlier relates to this point. In the end, SI through the SBI seeks to get an answer to *whether*-type questions (e.g., whether the fundamental space is homogeneous). In this context, symmetries of laws would shed light on that. But it seems to me, all this assumes that physical symmetries enter a physical theory, as it were, neutrally, in such a way that they do not contain any element that presupposes the answer to the whether-type question at stake. Yet, if all what I have been argued so far is right, and if the by-stipulation approach to symmetries is taken, then this is not true: in many cases, some of which I have explained, physical symmetries enter a physical theory to meet some standards of what is to be a good, acceptable physical theory. And this entails that physical symmetries enter a physical theory to reply to *how*-type questions. To take Dürr and Teufel’s example: under the assumption that the space is homogeneous, *how* can laws of nature be formulated “to follow this rule” (Dürr and Teufel’s expression)? But, as I have already argued, this clashes with SI’s metaphysical aspirations and the efforts to use symmetries to reply to whether-type questions. Once again, the risk of circularity and categorical mistake looms.

To finish, let me briefly put all the pieces together. **P1** in any instance of the SBI is taken as a formal premise, which just expresses that a set of laws turns out symmetric under a given transformation in a theoretical context. I have argued that such a simple, innocent statement already introduces non-trivial metaphysical content in the argument. This was the reason for examining it in more detail. My main point was that **P1** requires to fence in a validity domain, that is, to select some expressions of the laws (and some of their solutions) as the relevant ones. This means that **P1** mainly expresses a property (the symmetry) of such a validity domain. For it to work in a metaphysical argument, such a validity domain in turn requires to bear some metaphysical priority with respect to the rest of the theory domain. The validity domain where we find the symmetries is, within a physical theory, metaphysically prior to those where we find less (or none) symmetries. The simplistic, but confidently valid example of Eq. 1 and Eq. 2 showed the advantages and disadvantages of this extra assumption. So, this is the first conclusion –SBI, and the SI in consequence, need that **P1** expresses certain ontological content.

There may however be very good reasons for **P1** to express the content it does. In fact, the physical practice itself shows it –physical symmetries are in effect fenced in in certain validity domain for varied purposes. And this may indeed be given an ontological interpretation. At this point, I have drawn the attention towards how physical symmetries enter physical theories. In relying on the distinction between by-stipulation and by-discovery approaches to symmetries, I have argued that if the by-stipulation approach is given, then it clashes with SI and the SBI. In other words, the by-stipulation approach seems incompatible with taking physical symmetries as guides to ontology. To avoid this apparent incompatibility, some methodological principle (as Earman’s adequacy criteria) can be invoked. Nonetheless, I have suggested that in that case the principle in turn requires an ontological reading, which can be challenged by offering an epistemic-normative reading. The two most pressing problems now are the risks of circularity and categorical mistake.

## Conclusion

Most discussions gravitating around the philosophical value of physical symmetries in physics have so far put the efforts in delivering a substantial concept of physical symmetry that make the SBI work. In this paper, I have tried to shift the focus. I have rather centered in the formal premise of the inference, which states that a physical symmetry holds, calling for some philosophical caution when endorsing the SBI. My general point was that the fact that a physical symmetry holds within a physical theory comes along with a series of assumptions that may hamper the viability of the SBI, at least as how they are frequently employed in philosophy of physics and metaphysics of science. There is of course nothing wrong or precipitated in the physics. Physical symmetries are incredibly useful and a marvelous tool for theory developing. Neither is there anything particularly misguided about how physicists come to regard physical symmetries –a strong realist commitment about symmetries may heuristically motivate to push physics forward. My concern is how much philosophers should read off from them and to what extent we are allowed to believe that our long-standing metaphysical problems can be straightforwardly naturalized (via, for instance, the SBI) and eventually solved. I remain skeptic.

## Data Availability

“Not applicable”.

## Footnotes

1 Sklar (1974), Nozick (2001), Ismael and van Fraassen (2003), Brading and Castellani (2007), Healey (2009), and Dasgupta (2016) for a general analysis; for particular cases, see Horwich (1987), Price (1996), and Arntzenius (1997) for time-reversal invariance and time’s direction; Baker (2010) for natural quantities; North (2009, 2013) for geometrical structures in classical mechanics and quantum mechanics respectively; Allori et al. (2008) for interpretations of quantum mechanics.

2 By “fundamental reality” or “fundamental ontology”, I refer to those entities, properties, relations, or structures that are irreducible to anything else, and to which everything else reduces. Under SI, the best way to know which those fundamental, irreducible entities, properties, relations, or structures are is through physical symmetries.

3 I take for granted that the pairs a priori/necessity and a posterior/contingency go together. Yet, there would be two further relations I have left out: the pairs priori/contingency, on the one hand, and a posteriori/necessity, on the other. For this particular case, I do not see that any of these combinations yields a conceptually fruitful notion of physical symmetries. In particular, if symmetries were a posteriori and necessary, they would be *essences* in the Kripkean sense. As far as I know, nobody has defended such a view and I see no reason to think that symmetries may be essences. However, in general, Saul Kripke (1980) has persuasively argued for the existence of genuine cases. A proposition like “the length of stick S at time t0 is one meter” would be a priori and contingent (where “the length of S” is a non-rigid designator and “one meter” is so, being hence contingent; but it is obvious that the claim is knowable a priori at least for those users that stipulates the reference of “one meter”). A proposition like “gold is the element with atomic number 79” would be a posteriori and necessary (under the assumption that elements have essences, and it is a science’s task to discover them).

4 I offer a rather brief and informal presentation of the argument. An in-depth analysis and explanation can be found in Teller (1998), Martin (2002), Lyre (2004), Hetzroni (2021).

5 I thank an anonymous reviewer for drawing my attention to this criticism.

6 The idea that some physical symmetries are demanded in a physical theory has been motivated elsewhere. Lopez (2021b) argues that time-reversal invariance must be stipulated to work out the properties of the time-reversal transformation; Arntzenius and Greaves (2009) raise an akin point in classical electromagnetism, following the account assumed in textbooks. Peterson (2015) offers a broader view along with alternatives.

7 It might be claimed that Wigner’s view is too strong. Are there no natural laws in an Aristotelian world? Not necessarily. The argument, respecting Wigner’s spirit, can be slightly deflated and rephrased as following. The point is that *some* symmetries are required to distinguish between initial condition/laws of nature. In an Aristotelian world, there would not be some symmetries that we do have in, say, a Newtonian world (e.g., space-translation invariance), but there would be others (see Earman, 1989, Chapter 2.6, for some examples). The set of symmetries would be more restricted (and that’s why the laws of nature of an Aristotelian universe cannot be the laws of *classical physics*), but, in the line of Wigner’s word, it can be said that the Aristotelian laws of nature are what they are because there are some “Aristotelian” symmetries.

8 See references in fn. 6 for further examples.

## Code availability

“Not applicable”.
